# Dr. Hunt’s Case of General Hæmorrhagic Tendency; with Remarks by Dr. Reynell Coates

**Published:** 1842-07-23

**Authors:** R. T. Hunt

**Affiliations:** Surgeon of Manchester


### ANALECTA.

*Case of General Hæmorrhagic Tendency.* By R. T. Hunt, Surgeon of Manchester. (Hereditary Hæmorrhage?)—The injuries, of which the following communication contains an account, occurred to William Lawson, aged seven and a-half years. He had no peculiarity of appearance. He had brown hair, gray eyes, moderately clear but not florid complexion, but was at this time (Nov. 7, 1834,) thinner than formerly, apparently in consequence of recent severe hæmorrhage. He was always remarkably active and daring, and very passionate. His father, an engraver, had always experienced some difficulty in stopping the bleeding when his fingers were cut with the graver. He died of apoplexy a few months since. His mother, who has had a numerous family, is reported always to have suffered from hæmorrhage after labour. She states that when William was only a day or two old, such profuse bleeding occurred from the navel, that for many days he was not expected to live; that during the first year he suffered almost constantly from diarrhœa, and occasionally from tooth fever; and that when about two years old, he fell against the fender and cut his his head. The wound did not bleed much at the time, but the second night afterwards the bleeding increased so much that it was with great difficulty stopped by plaster and bandages.

February 3d, 1833. As he was sliding astride down the banister-rail, he slipped and fell, with his mouth open, against the end of the rail, and thus lacerated the inside of the upper lip near the fræenum, which was partially divided; the gums also were torn, and separated from the alveoli. It was an injury that would have caused considerable hæmorrhage from any individual, but the bleeding, for the first two days, was not of any consequence.

5. I saw him for the first time, the bleeding having become very profuse during the last night. There were coagula between the lips and teeth, continual general oozing of arterial-coloured blood from the injured surface, but no jet, nor any vessel bleeding separately, perceptible. A common astringent lotion was applied, and, afterwards, spirit. terebinthinæ, and argenti nitras in substance, by which means the bleeding was slightly checked, but returned at intervals until its effects upon the system had become truly alarming.

7. He was now quite blanched; his voice could scarcely be heard, nor could he raise himself in bed; his extremities were cool; his pulse quick, feeble, small, and irregular; and his pupils widely dilated. He would take nothing but a little milk and water, and that only occasionally, in consequence of his fearing that it would increase the bleeding. The actual cautery was now freely applied, with the effect of stopping the hæmorrhage, of which there was no recurrence; and in a few days he was sufficiently well to go out, though still much debilitated. The only medicine he took was a common aperient.

March 4. The skin over the left eyebrow was slightly grazed by a brass shoe-horn thrown at him by one of his brothers. The next day when I saw him, blood was constantly oozing from the wound, and no vessel large enough for a ligature perceptible. I applied a very thick compress of lint, supported by adhesive plaster and a bandage. In the morning the dressings were separated from the wound by a coagulum as large as a walnut, which had apparently thrust them off. The nitrate of silver was applied, the dressings replaced, and there was no more hæmorrhage.

April 3d, 1834. I again saw him in consequence of bleeding of the tongue: Two days before he had fallen over a wheelbarrow, and slightly bit his tongue in two places. There had been occasional bleeding till the night of April 2, when it became excessive, the mouth having been repeatedly filled with large coagula. The nitrate of silver in substance was first applied, and a strong alum lotion, the bleeding still recurring. The actual cautery was used three times during the day, a short cessation of the hæmorrhage following each application.

4. The bleeding having continued, the actual cautery was repeated, and lint soaked in spirit. terebinthinæ applied. The system now began to suffer very severely from the loss of blood. The same treatment was continued during the 5th and 6th.

7. The patient being now apparently sinking, two small pieces of wood were applied, one upon, the other under the tongue, and both posterior to the wounds. These pieces were connected at their extremities by ligatures, by means of tightening which, the tongue was forcibly compressed. This instrument was kept on for twelve hours, when the anterior part of the tongue had become swollen, inflamed, and almost gangrenous, in consequence of which the pieces of wood were removed. Eight hours afterwards the hæmorrhage returned, but was for some time checked by the application of lint soaked in spirit. terebinthinæ. The tongue having by this time recovered from the previous effects of pressure, a pair of spring forceps guarded with lint was applied, but caused so much pain as to render its removal necessary. The instrument, formerly mentioned, was again applied and retained for twelve hours, after which time the hæmorrhage, which had continued nine days after the accident, with the exception of the intervals recorded, fortunately did not return. No medicine was given until the cessation of the bleeding.

June 15, 1835. He was struck on the vertex with a stone, which caused an irregularly-triangular laceration, penetrating nearly to the cranium. There was immediately considerable bleeding, which could not be stopped by the usual dressings. On taking off the bandages his mother had applied, a large coagulum was found attached to the lacerated parts, from beneath which blood was continually oozing. After removing this coagulum, a tent of lint was firmly thrust into the wound, and secured in this position by means of strips of lint spread with white of egg and flour, which, in a short time, formed a compact hard covering, nearly as solid as a crab's shell. The following mixture was prescribed:—

Sulphate of soda, one ounce;
Water, six ounces.

To take an ounce every four hours.

16, 9 A. M. The bleeding had continued all night, and the wound presented nearly the same appearances as before. The dressings were renewed, and the dose of sulphate of soda doubled.

9 P. M. The wound bled during the afternoon, but not so freely. The sulphate has not yet acted upon the bowels. The nitrate of silver was applied to the wound, and the dressings replaced.

17. The sulphate of soda has produced several free alvine evacuations, and no more bleeding has occurred.

22. No further hæmorrhage. The dressings firmly adherent, and required the application of poultices for their removal.

This boy had never suffered from hæmorrhage of an idiopathic character from any of the mucous surfaces, nor from purpura or any peculiar cutaneous affection.

William Lawson's mother died, the family became dispersed, and after many fruitless inquiries, I at length, by chance, discovered that he had been admitted into the Manchester Bluecoat School; and, on applying to Mr. Greswell, the surgeon to that institution, he kindly informed me that Lawson had died in August, 1840, in consequence of convulsions supervening upon an abdominal affection, apparently caused by too freely eating of fruit, and bathing when in a state of perspiration. There was no post-mortem examination. During his residence in the school he had a slight injury of the hand, which was followed by very troublesome bleeding, similar to that produced by the accidents previously noticed.

*Remarks.*

The cases of hæmorrhagic tendency, which first attracted the attention of pathologists, will be found in the "London Medical and Surgical Journal" for 1808, in a communication from Dr. Otto, of Philadelphia, U. S. Upon reference to these and other analogous cases, it will be noticed that some time usually elapsed after the receipt of the injury, previously to the appearance of severe hæmorrhage, the wound not bleeding in the first instance more than similar wounds in other individuals. This is important; for if we compare the time at which adhesion generally takes place, I believe it will be found to agree with the period of the supervention of hæmorrhage in these instances, provided the injuries be of equal severity. In such hæmorrhagic cases, the process, which has been termed adhesive inflammation, appears not to be accomplished in a normal manner, although the vascular action of the injured parts is increased to an extent apparently sufficient for all the purposes of adhesion. The divided extremities of the vessels, instead of becoming agglutinated by the adhesive medium, not only remain pervious, but appear to acquire an increase in calibre, which allows them to pour out blood so freely as to give it the appearance of exuding from every point of the surfaces of the wound. Whether this irregular performance of the adhesive process depends upon the peculiar condition of the blood in such individuals, or upon some difference in the arrangement of their capillary system, is a question not to be very easily answered. The case I have recorded, is a proof that there is no deficiency in the coagulative power of the blood; the coagula having firmly adhered to the surrounding uninjured structures, although the oozing from the wounds was continuous.

The failure of local treatment until the bleeding had proceeded so far as to threaten a fatal termination, and the consideration that injuries occurring in structures differing so much in their organisation were, when of equal severity, always followed by equally profuse hæmorrhage, would incline me to depend upon general treatment. I do not consider the slight trial given to the sulphate of soda in Lawson's case as very conclusive when separately viewed. But it acquires value when considered in connection with the treatment pursued in Dr. Otto's cases. I may add, that I should be inclined to place the greater reliance upon its efficacy, in consequence of my having for many years observed the beneficial effects of various saline preparations in affections of the vascular system, in which these are not usually administered.—*Prov. Med. Jour.* May 28, 1842.

[The history of the peculiar predisposition to hæmorrhage, which forms the subject of Dr. Hunt's paper, is one of growing interest, from the increasing frequency of the accident, and the well known tendency of the affection to recur by inheritance. At page 344 of the present volume, we have extracted a similar and fatal case resulting from the extraction of a tooth ; adding some comments and further references.

We observe a strong resemblance between the personal description of Dr. Hunt's patient, and our own case of hæmorrhage from the extraction of a tooth, published in 1828, both in regard to physical and moral peculiarities. As there is reason to believe that this tendency is characteristic of a peculiar temperament, it is very desirable that its distinguishing marks should be carefully observed and noticed by those who meet with it in the course of practice; and, as there are several families in the United States in whom it is known to be very strongly marked, the opportunity cannot be long wanting. A very singular peculiarity in the hereditary course of the affection has been noticed by Professor Elsaesser and others. It appears to be chiefly, though not absolutely confined to males ; it frequently skips a generation entirely; and, in at least one instance, it has affected exclusively the male descendants in the female line. We should be glad to receive further information on these curious points, which, should the precepts of physiology ever be brought to exert their proper influence upon questions of matrimonial alliances, may obtain high practical importance.

From the whole evidence before the profession, it does not appear that the remedial measures hitherto essayed in such cases can claim any very powerful support. The statements of Dr. Otto, and succeeding observers, in favour of the exhibition of sulphate of soda, are not of great weight when critically examined. In mild cases, like that of Dr. Hunt, the hæmorrhage has often been arrested under circumstances in which the agency of the remedial measures is, at least, very doubtful; and in those of a more severe character, mechanical pressure and all the styptics, including the various caustics, have not produced more than a debateable and very temporary benefit. Dr. Hunt succeeded, on one occasion, with the actual cautery, and a very few other instances of similar success are on record; but, in the case narrated at page 344, not only did it fail, but an accidental touch of the iron to the lip of the patient established a new point of hæmorrhage; and in our own case, already referred to, the burning extended the bleeding surface; the discharge at first oozing through the sloughs, and finally flowing through the uninjured gum beyond the limits of the action of the iron, and the mechanical pressure superadded upon them.

Agreeing with Dr. Hunt in the belief that we must look to general treatment rather than the usual local measures for success, we again urge the opiate treatment upon the notice of the profession, for reasons which are stated in our original paper on the subject.] R. C.

